# Ethnic and sex differences in hepatic lipid content and related cardiometabolic parameters in lean individuals

**DOI:** 10.1172/jci.insight.157906

**Published:** 2022-04-08

**Authors:** Kitt Falk Petersen, Sylvie Dufour, Fangyong Li, Douglas L. Rothman, Gerald I. Shulman

**Affiliations:** 1 Department of Internal Medicine and; 2 Yale Diabetes Research Center, Yale School of Medicine, New Haven, Connecticut, USA.; 3 Novo Nordisk Foundation Center for Basic Metabolic Research, University of Copenhagen, Copenhagen, Denmark.; 4 Yale Center for Analytical Sciences, Yale School of Public Health, New Haven, Connecticut, USA.; 5 Department of Radiology and Biomedical Imaging, Yale School of Medicine, New Haven, Connecticut, USA.; 6 Department of Biomedical Engineering, Yale School of Engineering and Applied Science, New Haven, Connecticut, USA.; 7 Department of Cellular and Molecular Physiology, Yale School of Medicine, New Haven, Connecticut, USA.

**Keywords:** Hepatology, Metabolism, Glucose metabolism, Insulin, Obesity

## Abstract

**Background:**

Nonalcoholic fatty liver affects 25% to 30% of the US and European populations; is associated with insulin resistance (IR), type 2 diabetes, and increased cardiovascular risk; and is defined by hepatic triglyceride (HTG) content greater than 5.56%. However, it is unknown whether HTG content less than 5.56% is associated with cardiometabolic risk factors and whether there are ethnic (Asian Indian, AI, versus non-AI) and/or sex differences in these parameters in lean individuals.

**Methods:**

We prospectively recruited 2331 individuals and measured HTG, using ^1^H magnetic resonance spectroscopy, and plasma concentrations of triglycerides, total cholesterol, LDL-cholesterol, HDL-cholesterol, and uric acid. Insulin sensitivity was assessed using Homeostatic Model Assessment of Insulin Resistance and the Matsuda Insulin Sensitivity Index.

**Results:**

The 95th percentile for HTG in lean non-AI individuals was 1.85%. Plasma insulin, triglycerides, total cholesterol, LDL-cholesterol, and uric acid concentrations were increased and HDL-cholesterol was decreased in individuals with HTG content > 1.85% and ≤ 5.56% compared with those individuals with HTG content ≤ 1.85%, and these altered parameters were associated with increased IR. Mean HTG was lower in lean non-AI women compared with lean non-AI men, whereas lean AI men and women had a 40% to 100% increase in HTG when compared with non-AI men and women, which was associated with increased cardiometabolic risk factors.

**Conclusion:**

We found that the 95th percentile of HTG in lean non-AI individuals was 1.85% and that HTG concentrations above this threshold were associated with IR and cardiovascular risk factors. Premenopausal women were protected from these changes whereas young, lean AI men and women manifested increased HTG content and associated cardiometabolic risk factors.

**Funding:**

Grants from the United States Department of Health and Human Resources (NIH/National Institute of Diabetes and Digestive and Kidney Diseases): R01 DK113984, P30 DK45735, U24 DK59635, and UL1 RR024139; and the Novo Nordisk Foundation (NNF18CC0034900).

## Graphical abstract

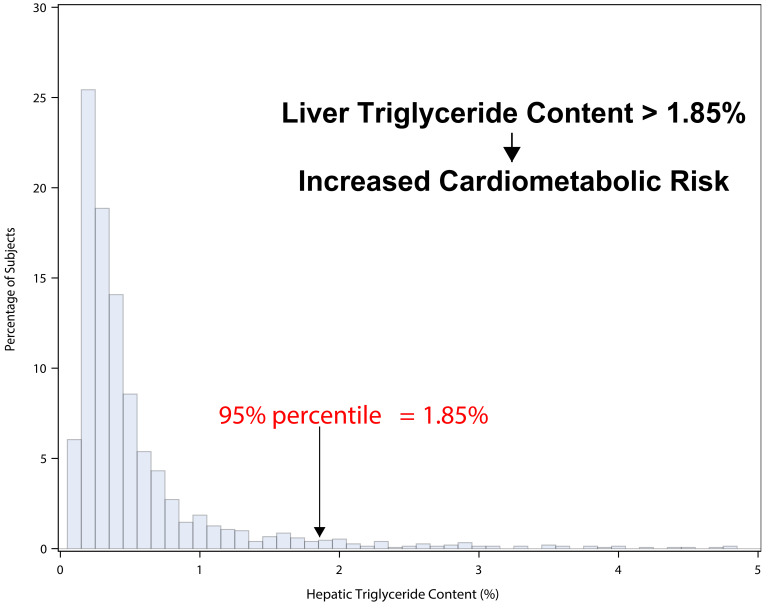

## Introduction

Nonalcoholic fatty liver (NAFL) is a major predisposing factor for the cardiometabolic syndrome (1–4), type 2 diabetes (T2D; refs. 5–7), nonalcoholic steatohepatitis (NASH; ref. 8), cirrhosis (9), and hepatocellular carcinoma (10–13). The gold standard for noninvasively assessing hepatic triglyceride (HTG) content is localized ^1^H magnetic resonance spectroscopy (MRS), and studies using this method or magnetic resonance imaging methods have demonstrated that NAFL affects between 20% and 30% of the US and European populations and can exist without abnormalities in markers of liver health such as liver enzymes (14–16).

In this regard the current diagnostic criterion for NAFL is based on the Dallas Heart Study, in which HTG content was measured using ^1^H MRS in a modified 1.5 T whole-body system in 345 individuals with a normal (<25 kg/m^2^) BMI (15). In this study Szczepaniak et al. reported that the distribution of HTG content had a median HTG content of 1.9% and a 95th percentile of 5.56% for the upper limit. Using this 95th percentile as the cutoff to define NAFL, the authors found that 30.7% of 2287 individuals had NAFL and that NAFL was more prevalent in men versus women, which they attributed to increased alcohol consumption in the men (17). However, most of their participants were overweight (33%) or obese (43%) and had a history of moderate to excessive alcohol consumption (~70%) and/or diabetes (~10%). Previous studies by our group and others have suggested that normal HTG content in lean individuals is below 3% (16, 18). Given that NAFL is found in lean individuals, even at the current definition of HTG greater than 5.56%, the questions of whether even lower HTG content is associated with cardiometabolic risks and insulin resistance (IR) and whether there are ethnic and/or sex differences in these parameters in lean individuals remain.

To examine these questions, we prospectively recruited 2331 mostly young, lean, healthy individuals and measured HTG, intramyocellular lipid (IMCL), and extramyocellular lipid (EMCL) content using localized ^1^H MRS. We performed a comprehensive metabolic characterization of each participant, including measuring concentrations of fasting plasma glucose, insulin, total cholesterol, triglycerides, HDL, LDL, aspartate aminotransferase (AST), alanine aminotransferase (ALT), and uric acid. In addition, we assessed IR in the overnight-fasted state, using the Homeostatic Model Assessment of Insulin Resistance (HOMA-IR; ref. 19), and in the postprandial state by performing oral glucose tolerance tests (OGTTs) to calculate the Matsuda Insulin Sensitivity Index (ISI; refs. 16, 20).

## Results

**Figure 1:**
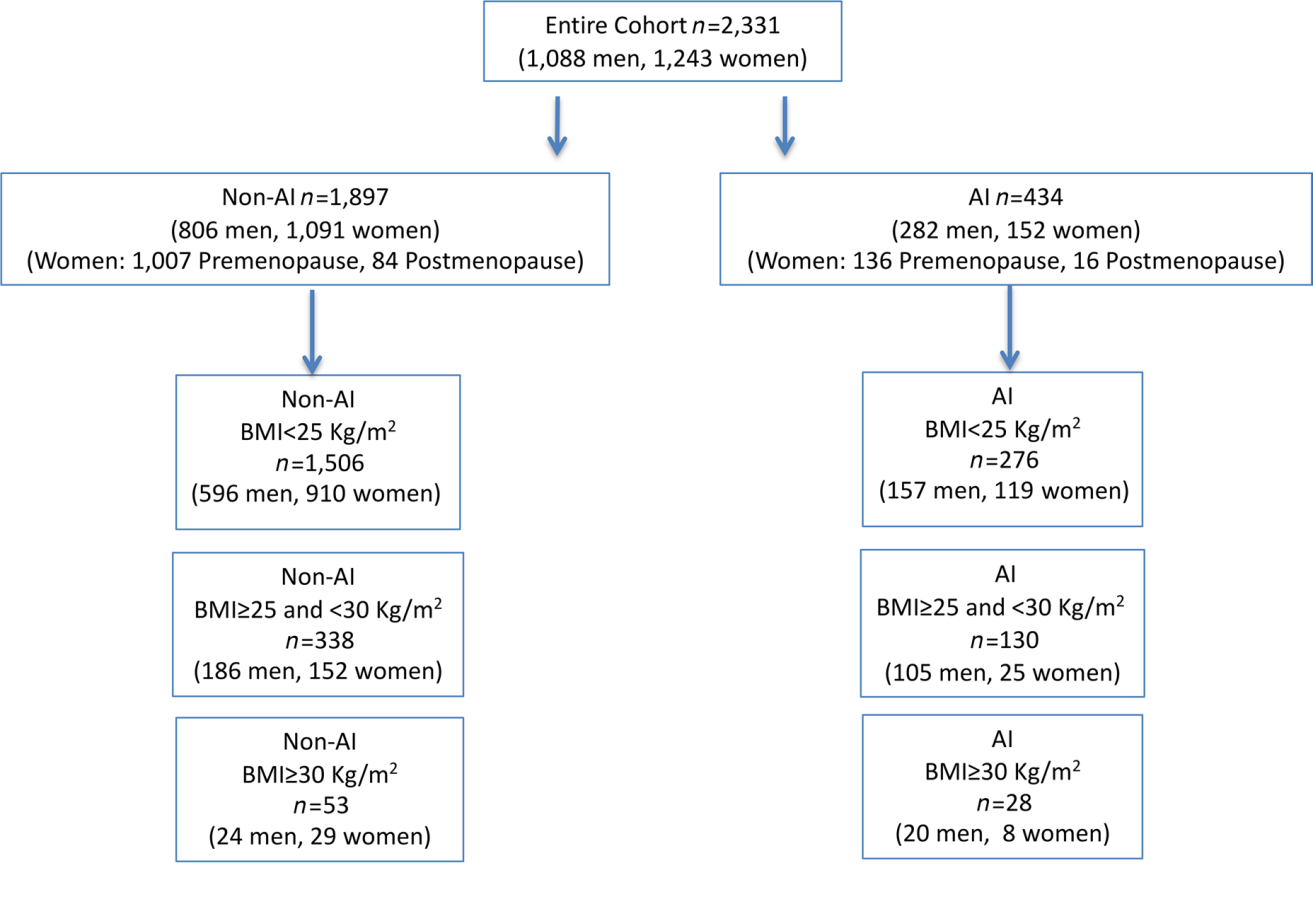
Flow diagram of recruited individuals and subgroups.

**Figure 2:**
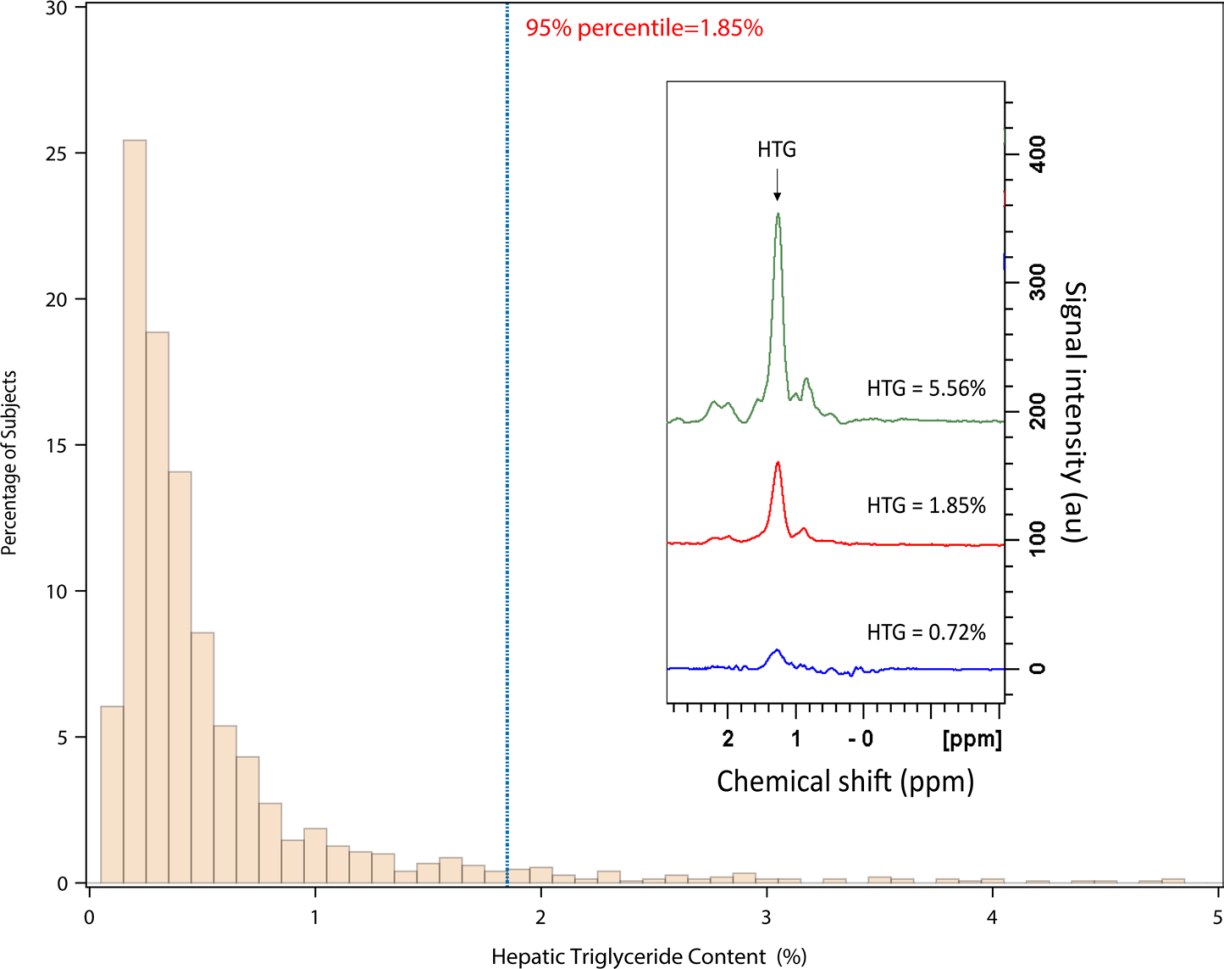
Distribution of HTG content in lean (BMI < 25 kg/m^2^) non-AI individuals (*n =* 1506). Consisting of *n* = 596 men, *n* = 910 women. Insert shows 3 typical ^1^H MRS spectra of HTG obtained at 4 T. ppm, parts per million.

Non-Asian Indian (non-AI) individuals were analyzed separately from the AI individuals, since, in earlier studies, we observed that the AI individuals as a group had a higher mean HTG compared with BMI-matched non-AI individuals (ref. 16 and Figure 1). We found that the distribution for HTG content in all these lean non-AI individuals (BMI < 25 kg/m^2^) was skewed to the right and had a median concentration of 0.35% and a 95th percentile of 1.85% (Figure 2). In order to determine whether HTG > 1.85% has any cardiometabolic related significance as compared with the current criterion of HTG > 5.56%, we compared fasting plasma markers of the cardiometabolic syndrome and IR between non-AI individuals with HTG ≤ 1.85% and with HTG content > 1.85% and ≤ 5.56% after adjusting for age, BMI, and sex. We found that fasting plasma concentrations of insulin, triglycerides, total cholesterol, LDL, and uric acid were all increased, and HDL was decreased in the individuals with HTG content > 1.85% and ≤ 5.56% compared with those individuals with HTG content ≤ 1.85% (Table 1). This group with HTG content > 1.85% and ≤ 5.56% also manifested increased diastolic blood pressure and whole-body IR as reflected by increased HOMA-IR and lower ISI compared with those with HTG content ≤ 1.85%. Similar differences in these parameters were also observed in lean individuals (BMI < 25 kg/m^2^) with HTG content > 1.85% and ≤ 5.56% compared with lean individuals with HTG content ≤ 1.85% (Supplemental Table 1; supplemental material available online with this article; https://doi.org/10.1172/jci.insight.157906DS1). Taken together these results demonstrate that HTG content > 1.85% and ≤ 5.56% is associated with IR and other risk factors for cardiometabolic disease.

To determine whether there are sex differences in HTG content and associated cardiometabolic parameters in non-AI individuals, we next compared these same parameters in men and women. We found that HTG content was significantly lower in women compared with men after adjusting for age and BMI and that this was associated with lower plasma concentrations of glucose, total cholesterol, LDL-cholesterol, and uric acid and increased concentrations of plasma HDL-cholesterol (Table 2). These changes were also associated with lower systolic and diastolic blood pressure in the women compared with the men.

Consistent with our prior study, we found that the mean HTG content was higher in the lean AI men compared with the lean non-AI men (Table 3 and ref. 16). Furthermore, this increased HTG in the lean AI men compared with the non-AI men was associated with increased fasting plasma glucose, insulin, and triglyceride concentrations; decreased HDL-cholesterol concentrations; and whole-body IR as reflected by increased HOMA-IR and decreased ISI (Table 3). Increased HTG content and IR in the lean AI men was also associated with increased IMCL and EMCL content as well as increased percentage of body fat.

We next examined whether in the AI population there are sex differences in HTG content and these same cardiometabolic parameters. Similar to the sex differences that we observed in non-AI individuals, we also observed an approximately 50% reduction of HTG in lean AI women compared with lean AI men (Supplemental Table 2), and this was associated with lower fasting plasma concentrations of glucose, triglycerides, and uric acid and increased plasma concentrations of HDL-cholesterol (Supplemental Table 2). These changes were also associated with reduced systolic and diastolic blood pressure in the AI women compared with the AI men.

Ethnic differences in HTG and these associated cardiometabolic parameters in lean AI women versus lean non-AI women were examined by comparing HTG content and the markers of cardiometabolic risk between lean AI women and non-AI women. Similar to the ethnic differences that we observed in lean AI men versus lean non-AI men (Table 3), we found that lean AI women had an approximately 1.5-fold increase in HTG as well as increased IMCL compared with lean non-AI women, which was associated with increased plasma insulin, triglyceride, and LDL-cholesterol concentrations and IR, as reflected by increased HOMA-IR and decreased ISI (Supplemental Table 3).

Given the observed sex-based reductions in HTG, IMCL, and IR in women (both AI and non-AI) compared with men (both AI and non-AI), we examined whether these protective effects wane with menopause. We found that HTG was 2.5-fold higher in postmenopausal women compared with premenopausal women and that this increase in HTG was associated with increased IMCL, EMCL, fasting plasma glucose, triglyceride, and total cholesterol, due mostly to increased LDL-cholesterol concentrations, and increased systolic and diastolic blood pressure (Table 4).

## Discussion

The current upper normal level of HTG is defined as 5.56% based upon a single study in 345 lean individuals where HTG content was measured by a whole-body ^1^H MRS 1.5 T system (15). In this study we examined a larger group of approximately 1500 healthy, lean, nonsmoking volunteers, who were carefully screened to exclude confounding factors such as excessive alcohol intake, any organ or systemic medical conditions, and any medications other than oral contraceptives, and characterized each participant using ^1^H MRS of liver, IMCL and EMCL content, laboratory tests, and an OGTT. We found that the 95th percentile of HTG content in healthy, lean individuals was only 1.85%, which was approximately 3-fold lower than the previously defined criterion for NAFL. The reason for this difference is unclear. It is possible that differences in the study inclusion criteria used by Szczepaniak et al. may account for these observed differences in HTG, because the group of 345 individuals studied is described only as being nonobese and nondiabetic, with normal liver function tests and minimal alcohol intake (15). Additional reasons for this discrepancy could be the large voxel size (27 cm^3^), used by Szczepaniak et al. in their studies, which potentially could include fat contamination from extrahepatic sites (e.g., subcutaneous fat, visceral fat, gallbladder, etc.), and the use of an assumed transverse relaxation time (T_2_) for all of their participants, which may overestimate the signal areas in the ^1^H MRS lipid spectrum.

To avoid these limitations, we assessed HTG content by ^1^H MRS in 4 relatively small liver volumes (15 mm^3^), which were carefully located to avoid nearby abdominal and subcutaneous adipose tissue. Furthermore, to further ensure proper volume location, we gated and synchronized our ^1^H MRS acquisition to the end of expiration to minimize the effects of movement during respiration. A water-suppressed lipid spectrum and a lipid-suppressed water spectrum were acquired in 4 different locations in each participant to account for liver inhomogeneity and the average value was used. In addition, the HTG content was corrected for T_2_ measured in each participant.

To determine whether HTG ≤ 1.85% would be meaningful as the revised cutoff for NAFL, we examined whether the group of non-AI individuals with HTG content > 1.85% and ≤ 5.56% were insulin resistant and whether they had any cardiometabolic related risk factors compared with individuals with HTG content ≤ 1.85%. We found that HTG between > 1.85% and ≤ 5.56% was accompanied by increased cardiometabolic risk factors as reflected by increased blood pressure, fasting plasma insulin, triglycerides, total cholesterol, LDL, and uric acid, and reduced HDL concentrations and IR, compared with non-AI individuals with HTG ≤ 1.85%. Thus, our findings show that HTG content > 1.85% but less than the current definition of NAFL of HTG > 5.56% is strongly associated with whole-body IR and increased cardiometabolic risk factors and that NAFL reflects more than a benign accumulation of lipid in hepatocytes.

We next examined whether there are sex differences in HTG and associated cardiometabolic risk factors in this cohort of healthy, mostly lean, young non-AI individuals and found that the mean HTG content was approximately 33% lower in lean non-AI women versus lean non-AI men. Furthermore, this reduction in HTG in the non-AI women was associated with reduced systolic and diastolic blood pressure; lower fasting plasma concentrations of glucose, LDL-cholesterol, and uric acid; and increased plasma HDL-cholesterol concentrations. Applying this new criterion (HTG ≤ 1.85%) for NAFL, we found that the prevalence of NAFL was 65% in overweight non-AI men, 52% in obese non-AI men, 35% in overweight non-AI women, and 48% in obese non-AI women.

Consistent with our prior studies, we found that healthy, lean AI men had an approximately 40% increase in HTG content and increased IMCL content compared with healthy, lean non-AI men (16). Furthermore, this increased HTG and IMCL content in the AI men was associated with increased fasting and 2-hour postprandial plasma glucose and insulin concentrations, increased HbA1c, as well as decreased plasma triglyceride and HDL-cholesterol concentrations and whole-body IR as reflected by increased HOMA-IR and decreased ISI. The cause of this difference is still unknown. However, we have shown that a modest reduction in body weight in this group of men was sufficient to significantly reduce HTG content from 14.0% to 3.8% (*P* = 0.05) (21) and improve the NAFL-associated dyslipidemia and IR, indicating that body weight is left-shifted in this ethnic group and that their BMI in general should be below 25 kg/m^2^, which currently defines leanness in non-AI (22). However, for comparison we still have applied the current definition of normal BMI < 25 kg/m^2^ to both groups and matched them for BMI.

We also examined whether there are sex differences in HTG content and cardiometabolic parameters in AI men versus AI women. Similar to the sex differences that we observed in non-AI individuals, we observed an approximately 50% reduction in HTG in lean AI women compared with lean AI men, and this reduction in HTG content in the AI women was associated with lower fasting and postprandial glucose concentrations, uric acid, AST, and ALT and increased plasma concentrations of HDL-cholesterol.

We next examined whether there were similar ethnic differences in HTG and these associated cardiometabolic parameters in AI women versus non-AI women. Similar to the ethnic differences that we observed in lean AI men versus lean non-AI men, we found that lean AI women had an approximately 2-fold increase in HTG content compared with lean non-AI women, and this increased HTG content was associated with increased plasma insulin, triglyceride, and LDL concentrations and IR as reflected by increased HOMA-IR and decreased ISI. Applying the criterion for NAFL of HTG ≤ 1.85%, we found that the prevalence of NAFL was 78% in lean AI men, 85% in overweight AI men, 86% in obese AI men, 22% in lean AI women, and 15% in overweight AI women.

We further examined whether these protective effects in women decrease with menopause and found that HTG was 2.5-fold higher in postmenopausal women compared with premenopausal women and that this increase in HTG was associated with increased cardiometabolic risk factors (increased fasting plasma glucose, insulin, triglyceride, and total cholesterol, due mostly to increased LDL concentrations, and increased systolic and diastolic blood pressure).

Taken together these results demonstrate both an ethnic and sex effect on HTG content and associated cardiometabolic risk factors where both AI men and AI women are prone to higher HTG content and increased IR and associated cardiometabolic risk factors than their respective non-AI sex and that the female sex promotes reduced HTG content and reductions in associated cardiometabolic risk factors.

The effects of sex on HTG content are controversial, with some studies showing lower prevalence of NAFL in women than men (15, 17, 22–26) but other studies showing increased prevalence of NAFL in women (27–29). In the Dallas Heart Study, Browning et al. observed higher HTG content in men than women, which they attributed to increased alcohol consumption in men (17). In this study, we found that women (both AI and non-AI) had decreased HTG content compared with men (both AI and non-AI). However, this reduction in HTG in women was independent of alcohol consumption, suggesting an important sex-related hormonal contribution to this phenomenon related to menopause. Consistent with this hypothesis, we found that HTG was 2.5-fold higher in postmenopausal women compared with premenopausal women and that this increase in HTG content was associated with increased IMCL and EMCL content and cardiometabolic risk factors. These data are consistent with rodent studies demonstrating that estrogen treatment in ovariectomized mice has a protective effect from developing high-fat diet–induced NAFL (30, 31).

In summary, here we show that the 95% upper limit for HTG content in young, lean, non-AI individuals was 1.85% and that HTG content above this value was associated with increased whole-body IR and increased cardiometabolic risk factors. Given the key role for NAFL and IR in the development of cardiometabolic disease as well as obesity-associated cancers, these results suggest that it will be important to develop new therapies in addition to caloric restriction (14, 21) for NAFL/NASH to achieve reductions in HTG content below 1.85% to reverse IR and/or prevent cardiometabolic events. Furthermore, we found significant sex and ethnic differences in HTG content in healthy, young, lean individuals. Understanding these associations between sex and ethnicity with NAFL will allow precision medicine to target specific groups to improve health, prevent disease, and reduce the rates of morbidity and mortality associated with NAFL and its associated pathologies (32).

## Methods

### Human participants.

From 2006 to 2020 we advertised in the local community and after an initial phone screen prospectively recruited and studied 2331 (1088 men and 1243 women) healthy, nonsmoking, sedentary individuals (not participating in regular physical activity, with a sedentary lifestyle, and on average walking <10,000 steps daily); with minimal history of alcohol consumption (less than 15 grams per day); and taking no medications (Figure 1).

Based upon our previous studies showing a higher prevalence of IR and hepatic lipid content in healthy, normal-weight AI men as compared with healthy, normal-weight eastern Asian, Black, White, and Hispanic individuals (16), each group were divided into non-AI individuals (*n* = 1897) and AI individuals (*n* = 434), all of whom were mostly between the ages of 25 to 35 years and lean. Ethnicity was self-reported (33). Each participant answered a questionnaire (34) about usual daily intake of food, alcohol use, eating habits, changes in body weight over the past 12 months, and physical activities. Habitual physical activity was measured over 3 consecutive days by a pedometer (Sportline, Inc.). The participants were also asked to describe the food and snacks consumed the day prior to the study visit, and the calorie count and composition of the diet for each participant were assessed. All participants underwent a complete medical history and physical examination, including measurement of HbA1c, to confirm that they were not diabetic. Blood pressure was measured using a Welch Allyn Vital Signs Monitor 300 Series in the supine position. A 3-hour OGTT (75 grams) was performed along with plasma insulin concentrations, which were measured at –10, –5, 0, 10, 20, 30, 60, 90, 120, and 180 minutes during the OGTT, to calculate HOMA-IR (35) and the ISI (20) as indices of whole-body insulin sensitivity.

### Analytical methods.

Plasma glucose concentrations were measured using a YSI 2700 STAT Analyzer. Plasma concentrations of insulin were measured using a double-antibody radioimmunoassay kit (Linco). Plasma triglyceride, total cholesterol, HDL-cholesterol, LDL-cholesterol, uric acid, AST, and ALT concentrations were measured enzymatically (Cobas Mira Plus, Roche Diagnostics Corp.).

### ^1^H MRS assessment of hepatic, intramyocellular, and extramyocellular lipid content.

HTG, IMCL, and EMCL contents were quantified by ^1^H MRS at 4 T (Bruker) as previously described (35). Briefly, HTG was measured by ^1^H respiratory cycle–gated STEAM spectroscopy in a 15 × 15 × 15 mm^3^ voxel. The acquisition was synchronized to the respiratory cycle and triggered at the end of expiration. A water-suppressed lipid spectrum and a lipid-suppressed water spectrum were acquired in 4 different locations to account for liver inhomogeneity and the average value was used. In addition, in each participant, HTG content was corrected for T_2_, using the transverse relaxation times of 22 ms for water and 44 ms for lipid. In each individual with a total HTG content above 4%, we measured T_2_ and used this correction to calculate the final HTG content (35). IMCL and EMCL contents were measured in the soleus muscle by ^1^H STEAM spectroscopy in a 10 × 10 × 10 mm^3^ voxel using an 8.5 cm diameter circular ^13^C surface coil with twin, orthogonal, circular, 13 cm ^1^H quadrature coils as described (35). Scout images of the lower leg were obtained to ensure correct positioning and to define an adequate volume for localized shimming using the FASTMAP procedure (35).

### Calculations.

The ISI was calculated from the plasma glucose and insulin concentrations before and during the OGTT as described (20). The ISI represents the composite whole-body insulin sensitivity, reflecting both hepatic and peripheral tissue insulin sensitivity. HOMA-IR was calculated according to this formula (36): (fasting plasma insulin [μU/L] × fasting plasma glucose [mg/dL])/(22.5 × 18).

### Statistics.

Variables are presented as mean ± SD or frequency and percentage as appropriate. The distribution of HTG in a subset of 1506 lean non-AI individuals was examined to obtain the 95th percentile as the cutoff of the normal value. A larger non-AI subset with the entire BMI range was classified as either HTG ≤ 1.85% or > 1.85% and ≤ 5.56%. Patient characteristics and 12-hour fasting cardiometabolic parameters were compared using general linear regression adjusting for age, BMI, and sex. Similar multivariate linear regression analyses were performed to examine group differences by sex, ethnic group, and age of women. Since HTG was right skewed, log transformation was conducted for group comparisons, and geometric mean and SD are presented. Statistical significance was set at *P* < 0.05, 2 sided, and all analyses were performed using SAS 9.4.

### Study approval.

Written informed consent was obtained from each participant. The study protocol conformed to the ethical guidelines of the 1975 Declaration of Helsinki as reflected in a priori approval by the Yale Institutional Review Board.

## Author contributions

KFP and GIS designed the experiments and wrote the manuscript with assistance from SD, FL, and DLR. KFP and SD performed the studies. FL performed all the statistical analyses. KFP, SD, FL, DLR, and GIS obtained and interpreted data.

## Supplementary Material

Supplemental data

ICMJE disclosure forms

## Figures and Tables

**Table 1:**
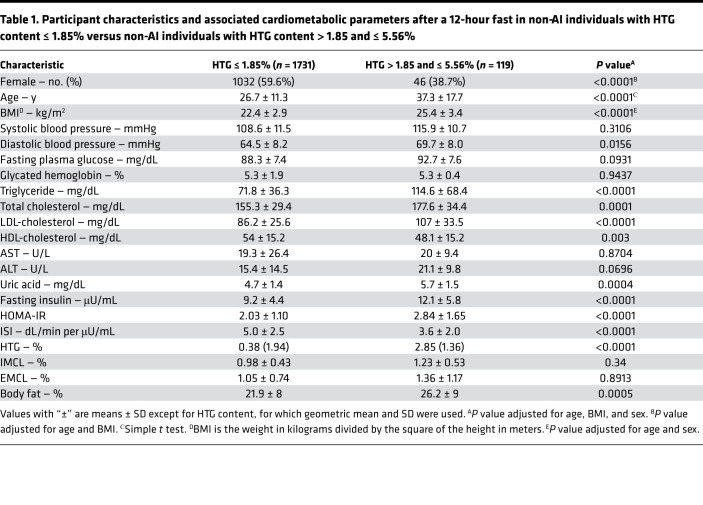
Participant characteristics and associated cardiometabolic parameters after a 12-hour fast in non-AI individuals with HTG content ≤ 1.85% versus non-AI individuals with HTG content > 1.85 and ≤ 5.56%

**Table 2:**
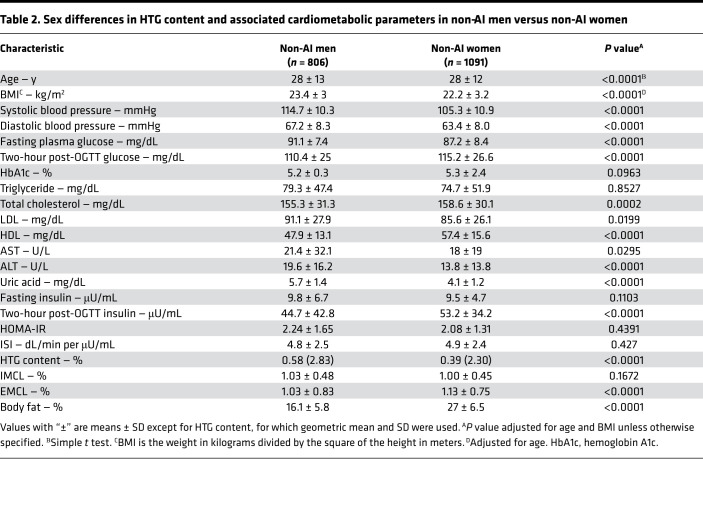
Sex differences in HTG content and associated cardiometabolic parameters in non-AI men versus non-AI women

**Table 3:**
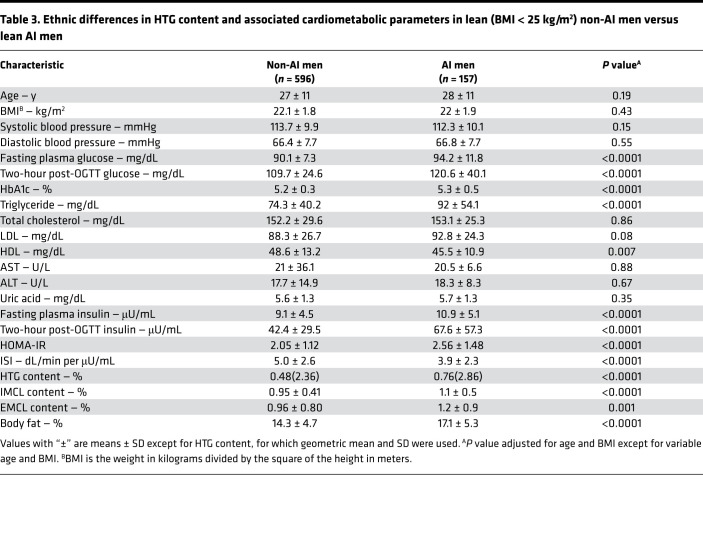
Ethnic differences in HTG content and associated cardiometabolic parameters in lean (BMI < 25 kg/m^2^) non-AI men versus lean AI men

**Table 4:**
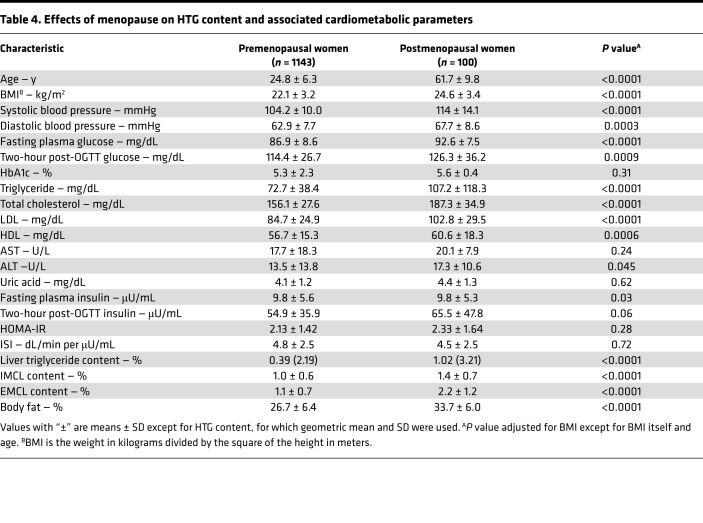
Effects of menopause on HTG content and associated cardiometabolic parameters

## Version 1 02/15/2022

In-Press Preview

## Version 2 04/08/2022

Electronic publication
