# Supplementary material for: Ethnic and sex differences in hepatic lipid content and related cardiometabolic parameters in lean individuals
Source: JCI Insight. 2022 Apr 8;7(7):e157906. doi: 10.1172/jci.insight.157906 (PMC9057590; doi:10.1172/jci.insight.157906)
Supplement: Supplemental data [file jciinsight-7-157906-s168.pdf]

Table S1. Participant characteristics and associated cardiometabolic parameters after a 12-hour fast in lean (BMI < 25 Kg/m^2) non-AI individuals with HTG content ≤1.85% vs. lean (BMI<25 Kg/m^2) non-AI individuals with HTG content >1.85 and ≤5.56%. *

Characteristic	HTG≤1.85% (N=1,431)	HTG>1.85 and ≤5.56% (N=67)	P-Value†
Age – Yr	26±11	35±17	<0.0001
Female – no. (%)	886 (61.9%)	22 (32.8%)	<0.0001
BMI – Kg/m^2 §	21.4±2	23.1±1.3	<0.0001
Systolic Blood Pressure – mmHg	107.6±11.1	114.2±9.8	0.03
Diastolic Blood Pressure – mmHg	63.8±7.9	68.9±6.7	0.002
Fasting Plasma Glucose – mg/dL	87.6±7.2	91.0±7.9	0.10
2-hr Post OGTT Glucose – mg/dL	112±24	121±30	0.007
HbA1c - %	5.3±2.1	5.2±0.4	0.84
Triglyceride – mg/dL	69.4±33	104.6±62.1	<0.0001
Total Cholesterol – mg/dL	153.9±28.5	169.1±35	0.09
LDL – mg/dL	84.4±24.6	99.8±34.2	0.02
HDL – mg/dL	54.8±15.4	48.2±16.8	0.0006
AST – U/L	19±28.3	20.6±11.8	0.77
ALT – U/L	14.7±14.7	20.9±9.8	0.02
Uric Acid – mg/dL	4.6±1.4	5.6±1.7	<0.0001
Fasting Plasma Insulin – µU/mL	8.9±4	10.6±5	0.001
2-hr Post OGTT Insulin – µU/mL	46.9±29.7	61.5±43.8	<0.0001
HOMA-IR	1.93±0.96	2.45±1.23	0.0003
ISI – dL/min per µU/mL	5.1±2.5	3.9±2.4	0.0003
Hepatic triglyceride content - %	0.35 (1.88)	2.76(1.35)	<0.0001
Intramyocellular lipid content - %	0.93±0.40	1.20±0.50	0.06
Extramyocellular lipid content - %	1.02±0.74	1.20±1.20	0.27
Body Fat - %	20.9±7.5	21.8±5.9	0.38

*Plus-minus values are means ±SD except for hepatic triglyceride content for which geometric mean and SD were used.
†P value adjusted for age and BMI except for age and BMI.
§Body mass index is the weight in kilograms divided by the square of the height in meters.

Table S2. Gender differences in hepatic triglyceride content and associated cardiometabolic parameters in AI men vs. AI women. *

**Characteristic**	**AI Men (Total N=282)**	**AI Women (Total N=152)**	**†*P-value***
Age – Yr	29.9±11.1	29.4±10.6	0.65
BMI – Kg/m^2 §	24.5±3.5	22.6±3.9	<0.0001
Systolic Blood Pressure – mmHg	114.3±10.3	102.8±9.0	<0.0001
Diastolic Blood Pressure –	68.6±8.1	63±7.5	<0.0001
Fasting Plasma Glucose – mg/dL	96±12	89.2±9.7	<0.0001
2hr-post OGTT Glucose – mg/dL	124.7±39.5	116.8±34.9	0.0352
HbA1c - %	5.4±0.5	5.3±0.4	0.3339
Triglyceride – mg/dL	109.0±70.5	83.2±51.3	0.009
Total Cholesterol – mg/dL	161.1±30.4	161±26.8	0.2764
LDL – mg/dL	98.9±27.9	90.8±23.4	0.09
HDL – mg/dL	44.0±11	54.8±15.5	<.0001
AST – U/L	21.8±7.1	16.9±5	<.0001
ALT – U/L	22.2±11.2	14.4±8.2	<.0001
Uric Acid – mg/dL	5.9±1.3	4.4±1.3	<.0001
Fasting Plasma Insulin – µU/mL	13.3±8.2	11.8±9.7	0.95
2hr post OGTT Insulin – µU/mL	80.7±71.1	74.4±49.7	0.77
HOMA-IR	3.21±2.56	2.63±2.12	0.52
ISI – (dL/min per µU/mL)	3.4±2.1	4.0±2.5	0.31
Hepatic triglyceride content - %	1.29 (3.68)	0.66(2.58)	0.0005
Intramyocellular lipid content - %	1.30±0.69	1.23±1.11	0.45
Extramyocellular lipid content -	1.27±0.83	1.30±0.94	0.39
Body Fat - %	21.0±6.2	29.8±7.3	<0.0001

*Plus-minus values are means ±SD except for hepatic triglyceride content for which geometric mean and SD were used.
†P value adjusted for age and BMI except for age and BMI.
§Body mass index is the weight in kilograms divided by the square of the height in meters.

Table S3. Ethnic differences in hepatic triglyceride content and associated cardiometabolic parameters in lean (BMI<25 Kg/m^2) non-AI women vs. lean AI women. *

Characteristic	non-AI Women (Total N=910)	AI Women (Total N=119)	†*P-value*
Age – Years	26.5±11	28.2±9.8	0.10
BMI – Kg/m^2 §	21.1±2	20.9±2.1	0.12
Activity – Miles/day	3.28±1.54	3.35±1.72	0.61
Systolic Blood Pressure -	104±10.2	101.1±8.2	0.002
Diastolic Blood Pressure -	62.6±7.6	61.9±7.0	0.32
Fasting Plasma Glucose -	86.3±6.9	86.6±6	0.89
2hr Post OGTT Glucose -	114.4±24.8	110.4±24.3	0.08
HbA1c - %	5.3±2.6	5.3±0.3	0.87
Triglyceride - mg/dL	69.4±34	77.7±38.3	0.03
Total Cholesterol - mg/dL	156.2±28.5	159.7±26.9	0.52
LDL - mg/dL	83.2±24.2	89.7±23.3	0.02
HDL - mg/dL	58.3±15.7	56.3±15.4	0.14
AST - U/L	17.8±20.3	17.1±5.3	0.76
ALT - U/L	13.2±14.2	14.6±8.8	0.35
Uric Acid - mg/dL	4.0±1.1	4.3±1.3	0.08
Fasting Plasma Insulin - μU/mL	8.9±3.9	11±10.4	<.0001
2hr Post OGTT Insulin - μU/mL	51.4±31.4	67.9±43.0	<.0001
HOMA-IR	1.92±0.96	2.3±2.1	0.0003
ISI - dL/min per μU/mL	5.1±2.4	4.4±2.6	0.007
Liver triglyceride content - %	0.34 (2.02)	0.53(2.34)	<0.0001
Intramyocellular lipid content -	0.94±0.41	1.2±1.2	0.0001
Extramyocellular lipid content -	1.09±0.73	1.2±0.9	0.19
Body Fat - %	25.3±5.3	27.3±5.5	0.002

*Plus-minus values are means ±SD except for hepatic triglyceride content for which geometric mean and SD were used.
†P value adjusted for age except for age itself.
Body mass index is the weight in kilograms divided by the square of the height in meters.
